# Supplementary material for: Transcriptomic Analysis on the Peel of UV-B-Exposed Peach Fruit Reveals an Upregulation of Phenolic- and UVR8-Related Pathways
Source: Plants (Basel). 2023 Apr 28;12(9):1818. doi: 10.3390/plants12091818 (PMC10180693; doi:10.3390/plants12091818)
Supplement: Supplementary file 1 [file plants-12-01818-s001.zip › Supplementary figures.pdf]

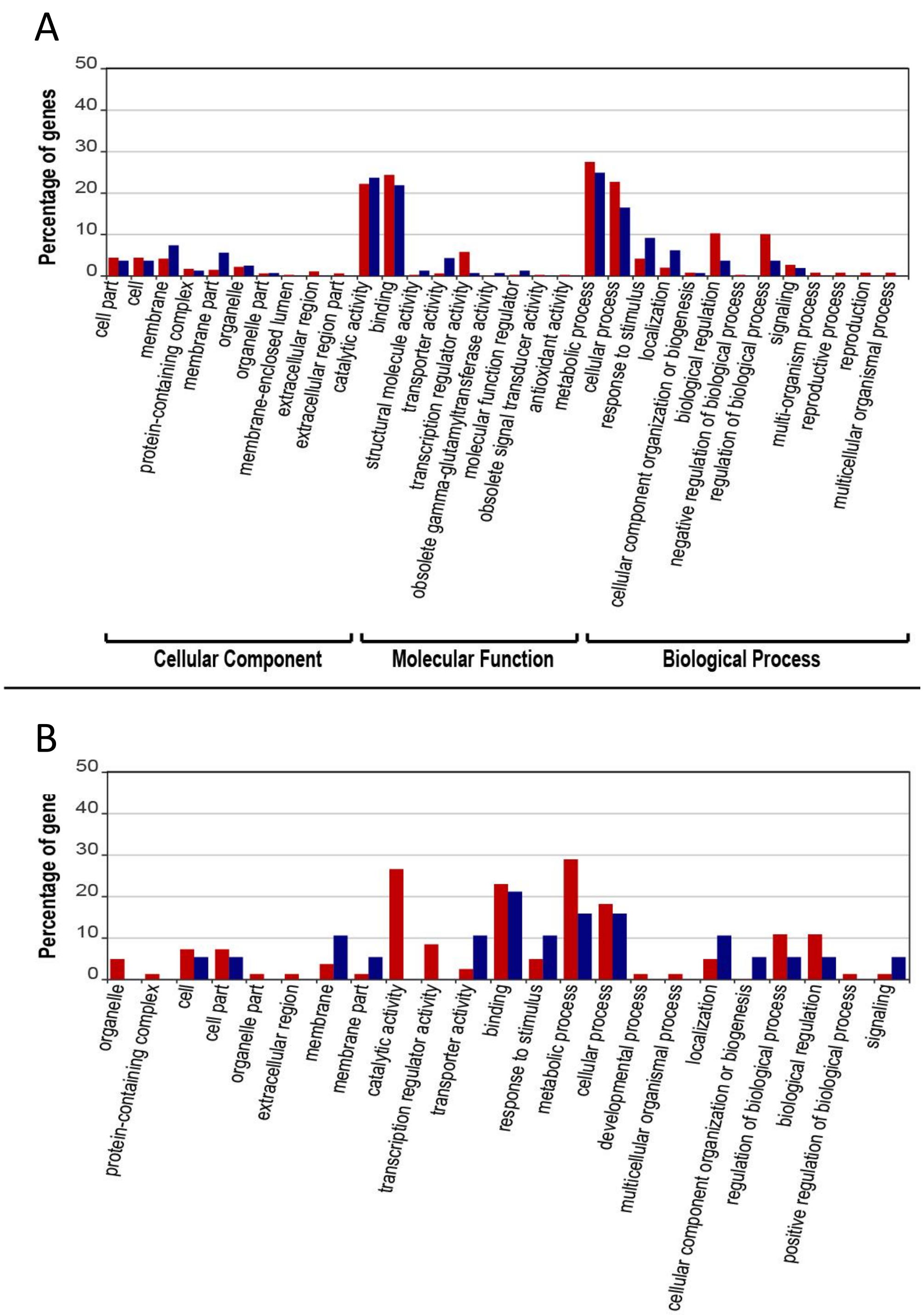

Figure S1. Distribution of GO terms for DEGs of peel of UV-B-treated peach fruit after (**A**) 1 h and (**B**) 3 h recovery time points. Over- expressed (red bar) and under-expressed (blue bar) GO terms are shown for each macro-category: “Cellular Component”, “Molecular Function”, and “Biological Process”.

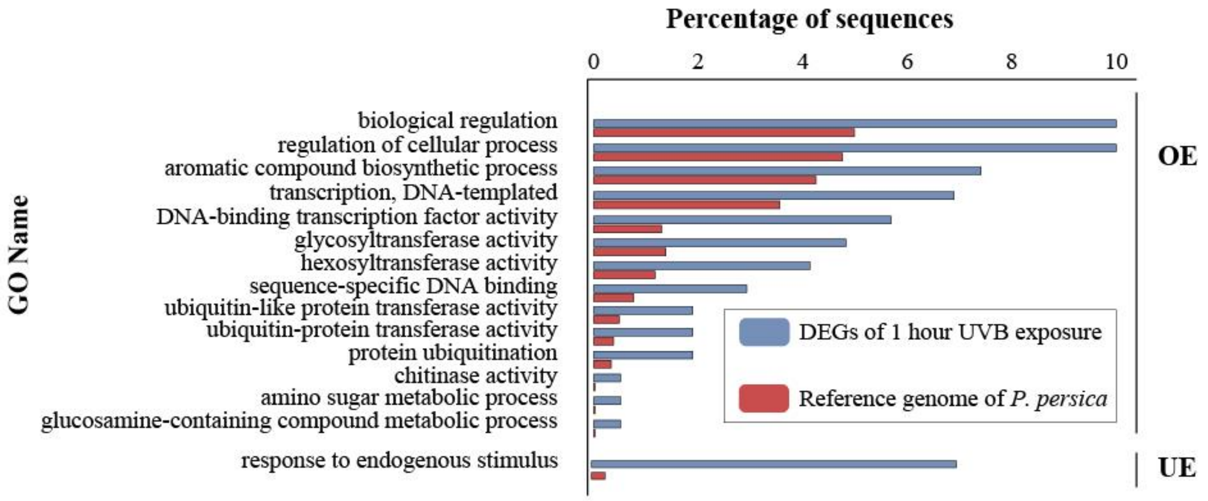

Figure S2. GO enrichment analysis for over (OE) and under (UE) expressed genes in the peel of UV-B-treated peach fruit after 1 h of recovery. Blue bars represent percentage of GO terms in DEGs. Red bars are the percentage of GO terms in reference *P. persica* transcriptome.

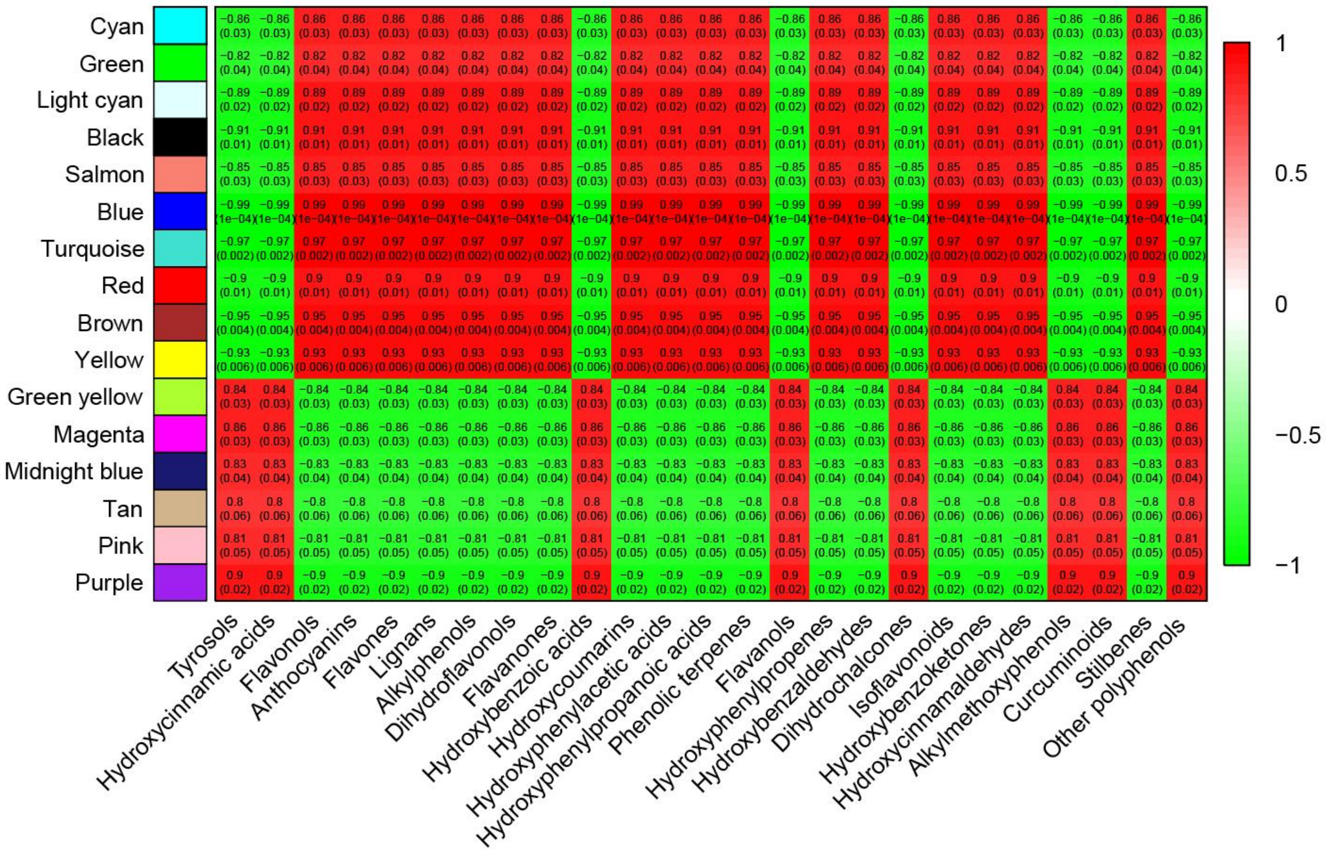

Figure S3. Gene network modules and biochemical trait correlation as calculated by WGNA. R^2 values and corresponding P-values are shown in each module-treatment comparison. Colour scale refers to R^2 and span from red (higher correlation) to green (lower correlation).
